# Molecular epidemiology of enteroviruses in young children at increased risk of type 1 diabetes

**DOI:** 10.1371/journal.pone.0201959

**Published:** 2018-09-07

**Authors:** Amir-Babak Sioofy-Khojine, Sami Oikarinen, Hanna Honkanen, Heini Huhtala, Jussi P. Lehtonen, Thomas Briese, Heikki Hyöty, on behalf of the TEDDY Study Group

**Affiliations:** 1 Department of Virology, Faculty of Medicine and Life Sciences, University of Tampere, Tampere, Finland; 2 Fimlab Laboratories, Pirkanmaa Hospital District, Tampere, Finland; 3 Faculty of Social Sciences, University of Tampere, Tampere, Finland; 4 Center for Infection and Immunity, Mailman School of Public Health, Columbia University, New York, New York; University of Minnesota College of Veterinary Medicine, UNITED STATES

## Abstract

**Background:**

Young children are susceptible to enterovirus (EV) infections, which cause significant morbidity in this age group. However, the current knowledge regarding the epidemiology of EVs and the circulating virus strains is mostly based on viruses detected in children with severe diseases leading to contact with the health care system, while the vast reservoir of EVs that circulate in the general population is less characterized.

**Methodology:**

The present study investigates the types and the prevalence of EVs circulating in the young children of the background population in Georgia, Colorado, and Washington State in the USA, and Germany, Sweden, and Finland in Europe.

A total of 4018 stool samples, collected monthly from 300 healthy and non-hospitalized children at the age of 3–18 months in 2005–2009, were analyzed for the presence of EVs using RT-PCR, followed by sequencing of the VP1-2A region of the viral genome to type the EV(s) present. All of the children carried type HLA-DQ2 or -DQ8 alleles associated with type 1 diabetes.

**Principal findings:**

Altogether 201 children (67%) were found to be EV positive. The prevalence was much lower in Finnish children (26%) than in the children of the other counties combined (75%). Infections increased by age and showed a nadir during the winter months. Children who carried both the HLA-DQ2 and -DQ8 alleles had less infections than children who were homozygous for these alleles. Coxsackieviruses type A were the most frequently detected viruses in all geographical regions. Coxsackievirus type A4, Echovirus type 18, and Echovirus type 25 were shed for longer time periods than the other EV types.

**Conclusions:**

Compared to prevalence data from symptomatic patients requiring medical attention, this study provides a better view of EVs circulating in young children in the USA and in Europe. The observations may prove useful for the selection of strategies for designing EV vaccines in the future. The study also confirms our previous serological findings suggesting that EV infections are relatively rare in Finland.

## 1. Background

Enteroviruses (EVs) are among the most common human viruses infecting nearly one billion people annually worldwide [1]. Even though most EV infections are asymptomatic or mild, EVs are responsible for a vast number of severe infections every year, including paralysis, encephalitis, aseptic meningitis, pleurodynia, pericarditis, myocarditis, hand-foot-and-mouth disease (HFMD), herpangina, severe neonatal systemic disease, and acute haemorrhagic conjunctivitis [2]. Young infants are susceptible to severe EV diseases, possibly due to their immature immune system.

EVs belong to the family *Picornaviridae* consisting of 15 species including *Enterovirus A-L*, and *Rhinovirus A-C* [3]. The EV species A, B, C and D infect humans, and they are traditionally divided into Poliovirus (PV) consisting of three serotypes, Coxsackievirus type A (CV-A) with 23 serotypes, Coxsackievirus type B (CV-B) with 6 serotypes, and Echovirus (E) consisting of 28 serotypes [4], as well as some more recently discovered numbered serotypes.

EVs show both endemic and epidemic patterns [5]. Epidemics are typically caused by certain EV types such as E-30 (causing aseptic meningitis), EV-D70 (causing acute hemorrhagic conjunctivitis), EV-A71 (causing severe central nervous system disease in the Far East), CV-A6 and CV-A10 (causing severe HFMD), and EV-D68 (causing flu-like symptoms) [1,6].

Recent advances in the molecular typing of EVs by sequencing have greatly improved our ability to study their epidemiology. These methods have also enabled the identification of EVs that are difficult to propagate in cell culture [7–9], and the accurate typing and classification based on genomic sequences [10–12]. A method introduced by Nix et al. [13] has been widely used for molecular typing of EVs. It uses a consensus degenerate hybrid oligonucleotide primer (CODEHOP) approach and amplifies VP1 coding region of all known EV serotypes [14].

Although these methods have advanced epidemiological studies in hospitalized patients, very little is still known about the epidemiology of EVs in the general population, where EV infections remain mainly asymptomatic or mild. These “hidden/subclinical” infections can have important health consequences, since they constitute a reservoir of EVs, which varies constantly due to recombination and a high mutation rate, thus leading to outbreaks and severe diseases in susceptible populations. In addition, some of these viruses may play a role in chronic diseases, such as type 1 diabetes (T1D) and cardiomyopathies, even if they do not cause any other apparent symptom [15,16].

## 2. Objective

The aim of this study was to characterize the large reservoir of EVs that circulate in young children in the general population of different geographical regions.

## 3. Materials and methods

### 3.1. Study population and collection of stool samples

The study subjects were selected randomly from the children who participated in a large birth-cohort study named “The Environmental Determinants of Diabetes in the Young (TEDDY)” recruiting children in six study centers in the USA and Europe. In the TEDDY study, large numbers of samples including blood and stool specimens are collected from children with genetically increased risk of T1D (19). Participants of the present study represent a randomly selected sub-cohort of TEDDY not enriched for any disease, sex or ethnic group. The participants were recruited from the general population and had no first-degree relatives with T1D. Study subjects carried one of the T1D-associated HLA-DQ genotypes that were screened for at birth (for further information see Hagopian et al. [17]). At the time of selection, all participants were non-diabetic and negative for T1D-associated autoantibodies.

A total of 4018 stool samples were collected monthly between the 17^th^ of January 2005 and the 16^th^ of March 2009 from 300 children including 147 girls and 153 boys aged 3-18 months, of whom 10 became positive for T1D autoantibodies and one child was eventually diagnosed with T1D (by the end of September 2016). The HLA-DQ genotypes were DQ2/8 (50% of the participants), DQ2/2 (27%) and DQ8/8 (23%). The distribution of the HLA types; however, differed across the study centers (S1 Table). It is worth mentioning that other HLA types, which pose an increased risk for T1D, were not included in this study. Altogether 20 (6.7%) children belonged to an ethnic minority (Hispanic, African American or other ethnic minority in the USA).

The sample series included 662 stool samples from 51 children in Colorado (COL), 669 samples from 50 children in Georgia (GEO), 668 samples from 50 children in Washington (WAS), 702 samples from 50 children in Finland (FIN), 645 samples from 49 children in Germany (GER), and 672 samples from 50 children in Sweden (SWE) as shown in Table 1.

**Table 1:** Number of stool samples and children studied, and EV positivity rates in different study centers.

Study center	Stool Samples			Children	
	N	Positive N (%*)	Typed N (%**)	N#	Positive N (%***)
COL	662	106 (16)	88 (83)	51	31 (61)
GEO	669	160 (24)	136 (85)	50	46 (92)
WAS	668	97 (15)	75 (77)	50	34 (68)
*Sub total*	*1999*	*368 (18)*	*299 (81)*	*151*	*111 (74)*
FIN	702	23 (3)	9 (39)	50	13 (26)
GER	645	93 (14)	79 (85)	49	36 (73)
SWE	672	107 (16)	92 (86)	50	41 (82)
*Sub total*	*2019*	*225 (11)*	*180 (80)*	*149*	*90 (60)*
Total	4018	586 (15)	479 (82)	300	201 (67)

* Percent of the samples

** Percent of EV positives, which are typed

*** Percent of the children positive for EV

The number of samples and the percentage where the type of EV was identified by sequencing are also shown. Study centers include Colorado (COL), Georgia (GEO), Washington (WAS), Finland (FIN), Germany (GER), and Sweden (SWE).

A median number of 13 samples were collected per child (range 2-16), and 99% of the children were sampled 8–16 times during the study period. Samples were collected evenly throughout the year in all centers (S2 Table) and their number did not differ significantly between the study sites (Table 1). Samples were collected at home by the parents and were sent to the TEDDY repository using express courier mail in the USA, or to local TEDDY centers in Europe using regular local mail. They were kept at an ambient temperature for one to three days during the shipment and temperature peaks during shipment were prevented by an ice-gel pack. Samples were stored at -80°C on arrival at the first destination.

Data on clinical symptoms were collected by parents at home using a diary and were translated to different symptom and disease codes (ICD-10 codes) by study nurses at each study visit. The details of the procedures used for the collection of symptom data have been described previously [18].

### 3.2. Ethics statement

The TEDDY study was approved by local U.S. Institutional Review Boards and European Ethics Committee Boards in Colorado’s Colorado Multiple Institutional Review Board, Georgia’s Medical College of Georgia Human Assurance Committee (2004–2010), Georgia Health Sciences University Human Assurance Committee (2011–2012), Georgia Regents University Institutional Review Board (2013–2015), Augusta University Institutional Review Board (2015-present), Florida’s University of Florida Health Center Institutional Review Board, Washington state’s Washington State Institutional Review Board (2004–2012) and Western Institutional Review Board (2013-present), Finland’s Ethics Committee of the Hospital District of Southwest Finland, Germany’s Bayerischen Landesärztekammer (Bavarian Medical Association) Ethics Committee, Sweden’s Regional Ethics Board in Lund, Section 2 (2004–2012) and Lund University Committee for Continuing Ethical Review (2013-present).

### 3.3. Enterovirus detection and typing

Stool suspensions (10% w/v) were prepared by thawing the samples and diluting them in Hanks balanced salt solution (HBSS, Sigma) containing 4% fetal calf serum (FCS). Suspensions were clarified by centrifugation at 500xg for 20 minutes at 4°C. Clarified supernatants were collected in aliquots in sterile cryo-preservation tubes and were stored at -80°C. EV RNA was extracted using a high-throughput nucleic acid extraction method (MagNa Pure extraction robot, Roche, Applied Science, Germany—using Total Nucleic Acid extraction kit, Roche). The viral genome was then detected by real-time RT-PCR as described elsewhere [19] using primers and probes shown in S3 Table. An average cycle threshold (Ct) from triplicate sample wells was used with a cut-off of 46 for positivity. All positive samples were further investigated by partial sequencing of a stretch of the VP1 coding region as described by Nix et al. [13].

In our study approximately 350-400 nucleotides were identified by sequencing, depending on the virus type. To identify the EV genotype, sequences were analyzed using “Enterovirus Genotyping Tool Version 0.1”; an online tool developed by the National Institute for public Health and Environment (RIVM), Bilthoven, the Netherlands [20].

### 3.4. Statistical analyses

The SPSS statistical package (version 22) was used for the analyses. The Kruskal-Wallis or Mann-Whitney U test was used to identify the statistical significance of the differences between groups. The Z-test was used to compare two proportions expressed as percentages. Binary logistic regression was used to analyze the probability of being EV positive according to the HLA-type, study center and sex of the child. The statistical significance was set to *p* = 0.05. The prevalence of infections was defined as the proportion of children with at least one EV positive stool sample (child positivity rate). The rate of sample positivity was defined as the proportion of EV positive samples. Infection episodes were defined by the detection of a solitary EV positive sample or the detection of the same EV strain in consecutive samples (identified by identical sequences). If the sequencing failed, Ct-values were used to identify the episode. An increase in the Ct-values in consecutively positive samples was assumed to represent one episode (indicating a decline in the virus shedding). An algorithm was created to calculate the length of an infection episode (S1 Fig).

## 4. Results

### 4.1. Prevalence of enterovirus infections

**Fig 1:**
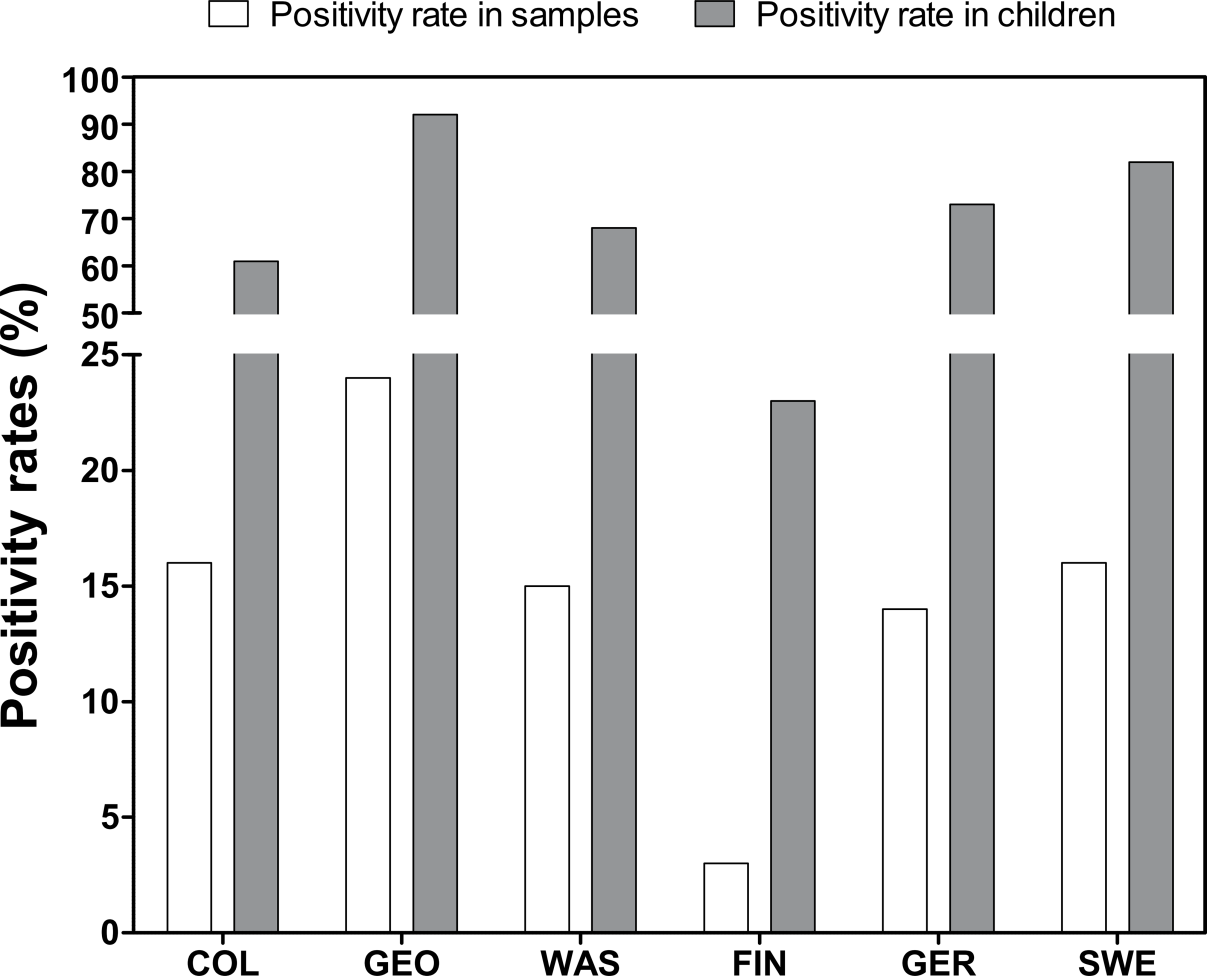
Prevalence of EVs represented as the child positivity rate and sample positivity rate in each study center. The difference in positivity rates between Finland and all other study centers is statistically significant in each category (*p*<0.001). Study centers include Georgia (GEO), Washington (WAS), Colorado (COL), Finland (FIN), Sweden (SWE) and Germany (GER).

Altogether 14.6% of all samples were EV positive by real-time RT-PCR using a cut-off of Ct = 46 (the majority of them were still EV positive using a lower cut-off of Ct = 42 being positive in 14.1% of total samples, S2 Fig). Sample positivity rate was the lowest in Finland (3.3%) and the highest in Georgia (24%, Fig 1). The mean Ct-value of positive samples (N = 586) was 31.5 (range 15.7–45.6, SD 6.8) showing no difference between the centers (*p* = 0.54).

At least one EV positive stool sample was detected in 67% of the study participants. The proportion of EV positive children was much lower in Finland than in the other countries combined (26% vs. 75%, *p*<0.001; Fig 1). The highest rates were seen in Georgia, where 92% of the children were EV positive. The prevalence of EV infections increased with the age of the child (S3 Fig). The mean number of positive samples per child was 1.98 (SD 2.05, Median of 1, interquartiles of 0, 1, and 3).

**Fig 2:**
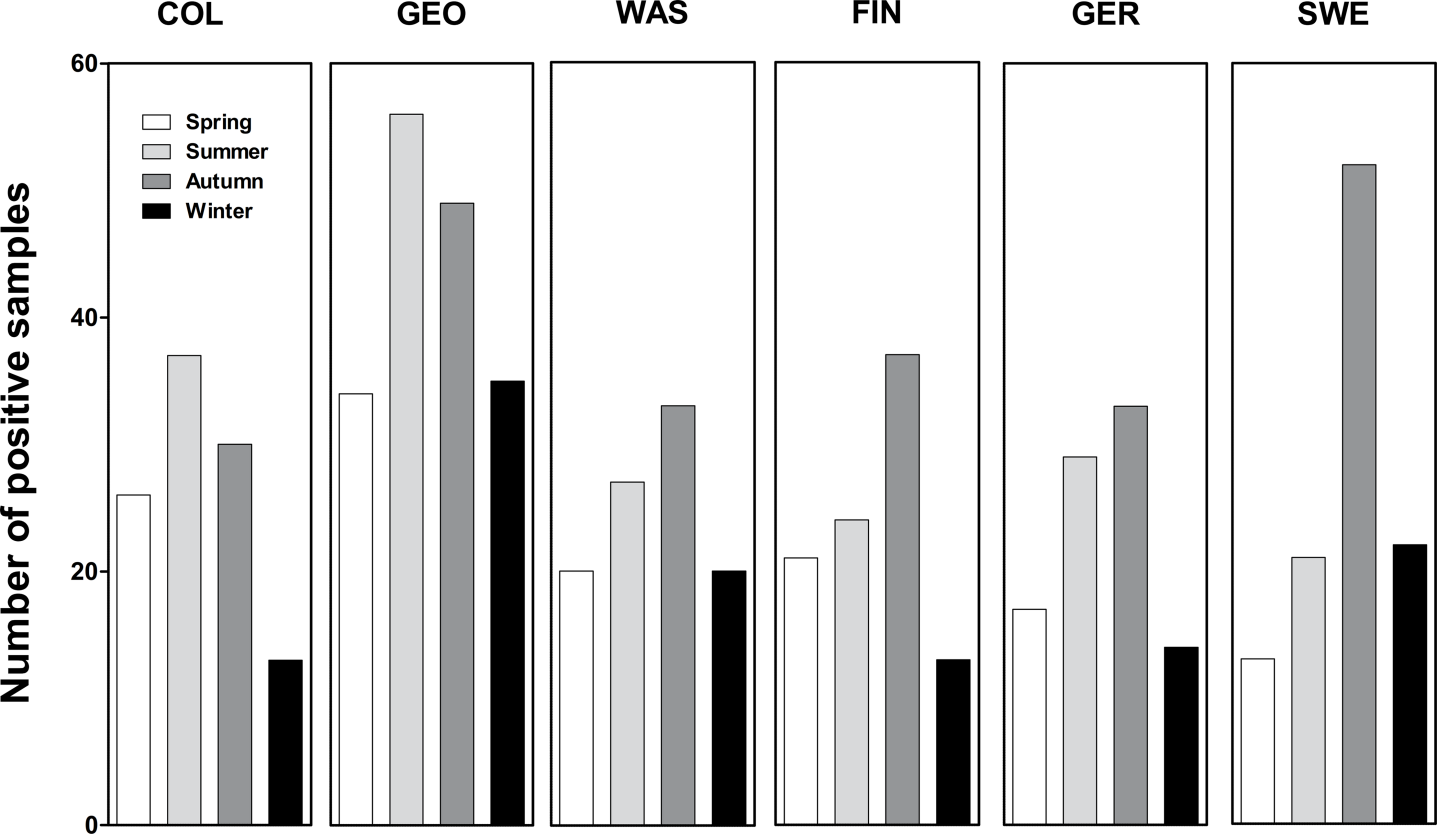
Seasonal distribution of EV-positive stool samples in different study centers. The year has been devided into 4 seasons from March to February (Spring includes March, April and May). The total number of EV-positive samples in each season is shown for each study center.

A clear seasonal pattern was seen in all geographical regions showing a peak during spring, summer, and autumn months (March-October) with some variation between the study centers (Fig 2). Sample positivity rate peaked in certain years. Colorado had a significantly higher number of EVs in 2007 (25%) compared to the average of 9.5% for other years (*p*<0.001). Georgia had a significantly higher number of EVs in 2008 (30%) compared to 21% for all other years (*p* = 0.018).

### 4.2. Prevalence of different enterovirus types

Typing was successful in 82% of the EV-positive samples (479 samples; Table 1). The success rate of genotyping depended on the virus load, since the typed samples had significantly lower average Ct-values compared to un-typed samples (mean Ct-value 30 versus 37, *p*<0.001). The majority (61.5%) of the genotyped viruses belonged to the species *Enterovirus A* (EV-A), 38% to *Enterovirus B* (EV-B) and only 0.5% to *Enterovirus C* (EV-C) (Table 2). Interestingly some human rhinoviruses (HRVs) were also amplified by the sequencing primers, because of their sequence homology with EVs (six samples); these were excluded from the analysis.

**Table 2:** Type of EVs in each study center and the rank of the virus among all typed viruses.

		Number of typed viruses in stool samples (Ranks)									% of typed viruses
Species	Genotype	USA	COL	GEO	WAS	Europe	FIN	GER	SWE	Total	
EV-A	CV-A2	31 (2)	9 (2)	17 (1)	5 (4)	6 (7)	1 (2)	3 (5)	2 (8)	37 (3)	9.6
	CV-A4	48 (1)	22 (1)	12 (2)	14 (1)	22 (1)	3 (1)	9 (1)	10 (1)	70 (1)	18.2
	CV-A5	18 (4)	2 (6)	5 (7)	11 (3)	12 (3)	0	5 (3)	7 (3)	30 (4)	7.8
	CV-A6	29 (3)	6 (3)	11 (3)	12 (2)	17 (2)	1 (2)	7 (2)	9 (2)	46 (2)	11.9
	CV-A8	2 (13)	0	2 (10)	0	2 (11)	0	1 (7)	1 (9)	4 (15)	1.0
	CV-A10	12 (5)	1 (7)	7 (5)	4 (5)	10 (4)	0	5 (3)	5 (5)	22 (5)	5.7
	CV-A16	10 (7)	2 (6)	6 (6)	2 (7)	5 (8)	0	1 (7)	4 (6)	15 (7)	3.9
	EV-A71	3 (12)	0	2 (10)	1 (8)	9 (5)	0	3 (5)	6 (4)	12 (10)	3.1
	Sub total	153	42	62	49	83	5	34	44	236	61.5
EV-B	CV-A9	5 (10)	0	5 (7)	0	6 (7)	0	2 (6)	4 (6)	11 (11)	2.9
	CV-B1	12 (5)	5 (4)	3 (9)	4 (5)	2 (11)	0	2 (6)	0	14 (8)	3.6
	CV-B2	8 (8)	1 (8)	6 (6)	1 (8)	2 (11)	0	0	2 (8)	10 (12)	2.6
	CV-B3	5 (10)	2 (6)	2 (10)	1 (8)	8 (6)	0	5 (3)	3 (7)	13 (9)	3.4
	CV-B4	8 (8)	4 (5)	3 (10)	1 (8)	8 (6)	0	7 (2)	1 (9)	16 (6)	4.2
	CV-B5	4 (11)	0	4 (8)	0	5 (8)	0	0	5 (5)	9 (13)	2.3
	E-3	4 (11)	0	4 (8)	0	0	0	0	0	4 (15)	1.0
	E-6	6 (9)	0	4 (8)	2 (7)	4 (9)	0	1 (7)	3 (7)	10 (12)	2.6
	E-9	1 (14)	0	0	1 (8)	3 (10)	0	2 (6)	1 (9)	4 (15)	1.0
	E-11	4 (11)	2 (6)	1 (11)	1 (8)	9 (5)	0	5 (3)	4 (6)	13 (9)	3.4
	E-13	2 (13)	0	2 (11)	0	6 (7)	0	4 (4)	2 (8)	8(14)	2.1
	E-18	11 (6)	5 (4)	3 (9)	3 (6)	1 (12)	0	1 (7)	0	12 (10)	3.1
	E-21	0	0	0	0	1 (12)	0	0	1 (9)	1 (16)	0.3
	E-25	12 (5)	2 (6)	9 (4)	1 (8)	1 (12)	0	0	1 (9)	13 (9)	3.4
	E-30	5 (10)	0	4 (8)	1 (8)	3	0	2 (6)	1 (9)	8 (14)	2.1
	Sub total	87	21	50	16	59	0	31	28	146	38
EV-C	CV-A1	0	0	0	0	1 (12)	0	0	1 (9)	1 (16)	0.3
	CV-A22	1 (14)	0	1 (11)	0	0	0	0	0	1 (16)	0.3
	Sub total	1	0	1	0	1	0	0	1	2	0.5
	Total	241	63	113	65	143	5	65	73	384	100
		Number of typed group of viruses in stool samples (%)									
	All CVA	156 (64.7)	42 (66.7)	66 (58.4)	48 (73.8)	81 (56.6)	5 (100)	33 (50.8)	43 (58.9)	237 (61.7)	-
	All CVB	37 (15.4)	12 (19)	18 (15.9)	7 (10.8)	25 (17.5)	0	14 (21.5)	11 (15.1)	62 (16.1)	-
	All EVs	45 (18.7)	9 (14.3)	27 (23.9)	9 (13.8)	28 (19.6)	0	15 (23.1)	13 (17.8)	73 (19)	-

Typed viruses are categorized in species EV-A, EV-B, and EV-C. Each child can be positive for a number of EVs. The number of each virus type is shown for each study center. The data from the USA, Europe, and the total numbers are shown separately for comparison. The last column shows the percentage of each virus (and species) among all typed viruses. Numbers in the brackets represent the rank of the virus among all viruses in each category (column). At the bottom 3 rows of the table the total number (and the percentage) of CVA, CVB, and EVs are presented.

CV-As were the most frequently detected EVs in all study centers. 37.7% of all children experienced at least one CV-A infection and 61.7% of all genotyped EVs were CV-As (Table 2). The second most common viruses were echoviruses (Es) that were detected in 16.3% of the children and 19% of the genotyped samples, followed by CV-Bs that were detected in 14.7% of the children and 16.1% of the genotyped samples (Table 2). Of the individual EVs CV-A4 was the most common virus in the stool samples (18.2% of all typed EVs) followed by CV-A6 (11.9%), CV-A2 (9.6%), CV-A5 (7.8%), and CV-A10 (5.7%; Table 2), a pattern which showed only minor variations between different centers. Only 3.1% of all typed EVs were EV-A71 and no EV-D68 was found. However, due to it's biological properties, EV-D68 is hard to detect in stool samples. Among different study centers, Germany had the most frequent CVBs (22% of the samples), followed by Colorado (19%), Georgia (16%), Sweden (15%), and Washington (11%) (Table 2).

EVs were detected rarely before the age of 6 months becoming more frequent at older age (S6 Table) and certain CAVs (CVA2, 4, 5, 6) showed an epidemic pattern peaking in certain years (S6 Table).

None of the children in this study had meningitis, myocarditis, encephalitis, paralysis or other severe symptoms typical for EV infection. Only 1/95 children with EV-A infection had symptoms compatible with HFMD (commonly caused by CV-A6, A10, A16 or EV-A71) at the time of the infection episode; the child was positive for CV-A6.

#### Duration of virus shedding

**Fig 3:**
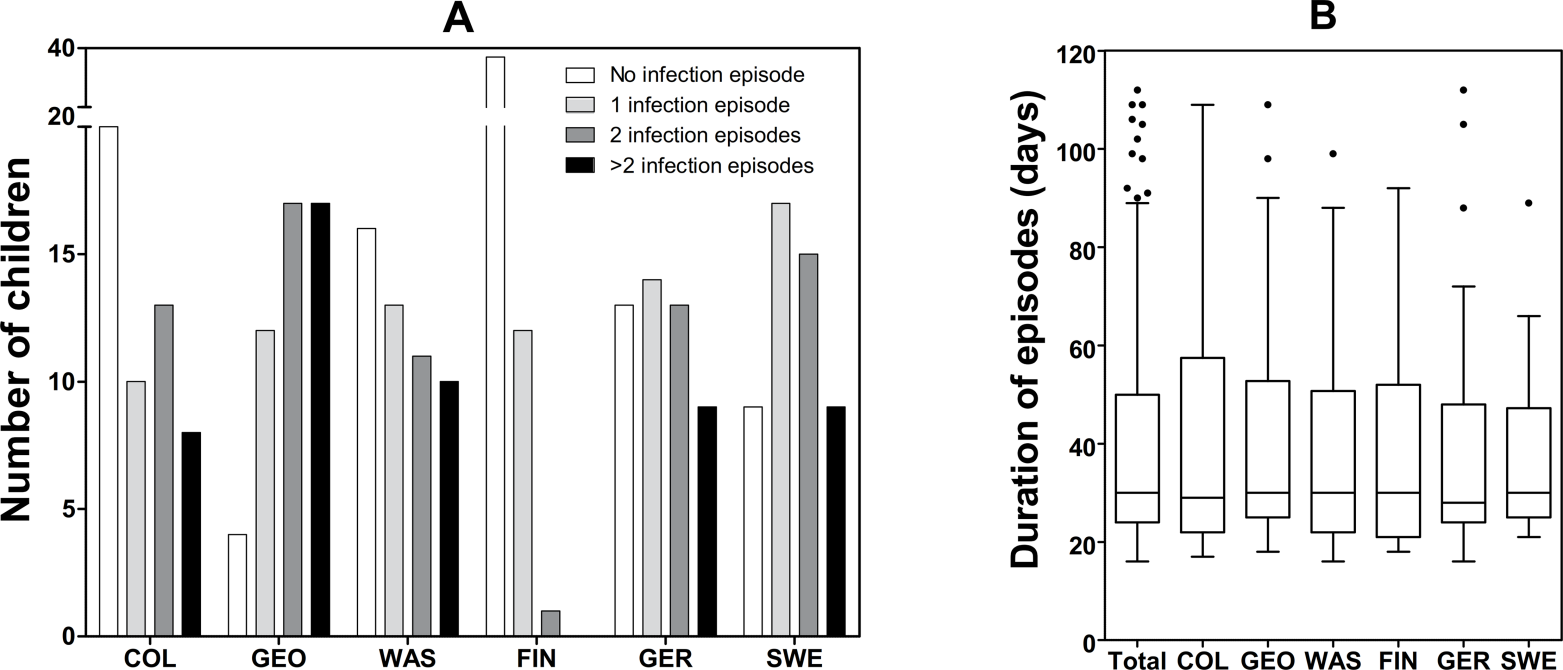
**The number of EV infection episodes (A) and the duration of episodes (B) in different study centers.** The number of infection episodes is shown by the number of the subjects having 0, 1, 2, or more than 2 infection episodes per child (A) and the duration of the episodes is shown in days (B). The thick horizontal lines in the boxes represent the median for each center with the inter-quartile values; the circles represent the outliers (B).

A total number of 411 EV episodes were identified using the criteria described in S1 Fig, of these 315 had a positive identification of the virus genotype. Altogether 268 (65.5%) of the episodes included only a single EV positive sample, 112 (27.3%) had two, 25 (6.1%) three, and 6 (1.5%) had four consecutive positive stool samples. At least one infection episode was observed in 164 children (55% of total participants) and 71 (43%) of them had only one infection episode, 51 (31%) had two episodes, 28 (17%) had three episodes, 12 (7%) had four episodes (7.3%), and 2 (1%) had five episodes. Finnish children had significantly less episodes than children in other countries (*p*<0.001, Fig 3). The mean duration of the infection episodes was 38 days (SD 20.4 days, median 30 days, interquartiles 24, 30, and 50) showing no difference between the study centres (*p* = 0.08, Fig 3).

**Fig 4:**
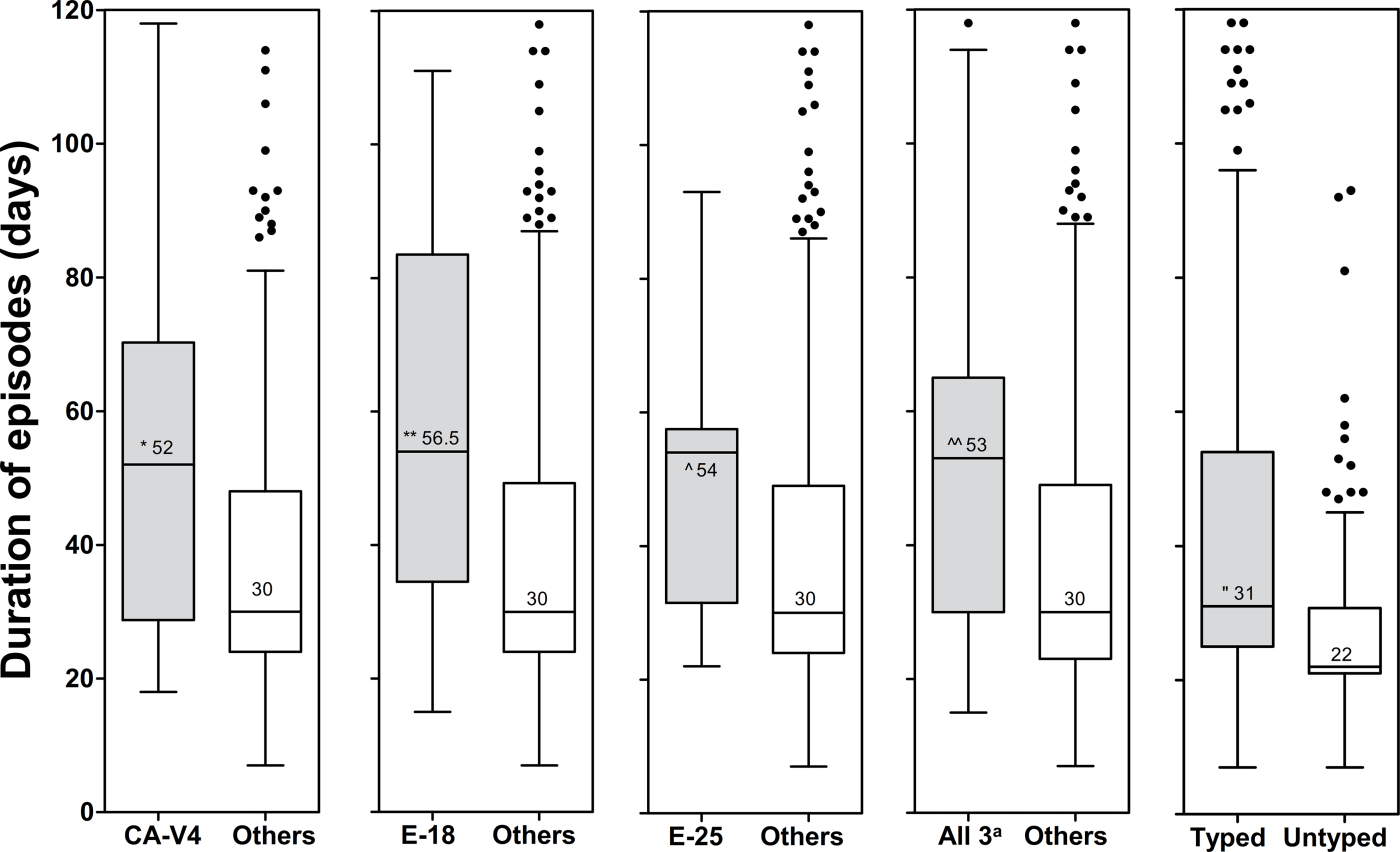
Duration of EV infection episodes caused by different EV serotypes compared to all other episodes. The duration of CV-A4 infection episodes was significantly longer compared to all other enterovirus types together (*p<0.001). E-18 and E-25 also had longer episodes (**p = 0.026 and ^^^*p* = 0.026). The three viruses together (CV-A4, E-18, and 25) had significantly longer infection episodes compared to the rest of the viruses (^^^^*p*<0.001). The length of infection episodes by un-typed enteroviruses was significantly shorter compared to typed viruses (^“^*p*<0.001). All 3^a^ refers to CV-A4, E-18 and E-25 together.

Among the successfully genotyped EV episodes CV-A4 episodes (N = 52) were characterized by significantly longer virus shedding time compared to all other EV types (mean 51 vs. 36 days, *p*<0.01; Fig 4). Similarly, E-18 episodes (N = 9) were longer compared to all other EVs (mean 59 vs. 36, *p* = 0.026), and the same was true for E-25 (N = 9; mean 47 vs. 38 days, *p* = 0.026; Fig 4). On the other hand, the episodes caused by un-typed EVs were significantly shorter compared to the episodes caused by typed EVs (mean 28 vs. 41 days, *p*<0.001). This may be a result of timing of sample collection (recovery phase of the infection) or the low levels of the virus shed in the un-typed episodes. Corresponding frequencies of EV episodes caused by different EV genotypes are shown in S4 Table.

### 4.3. Effect of host factors on enterovirus infections

**Fig 5:**
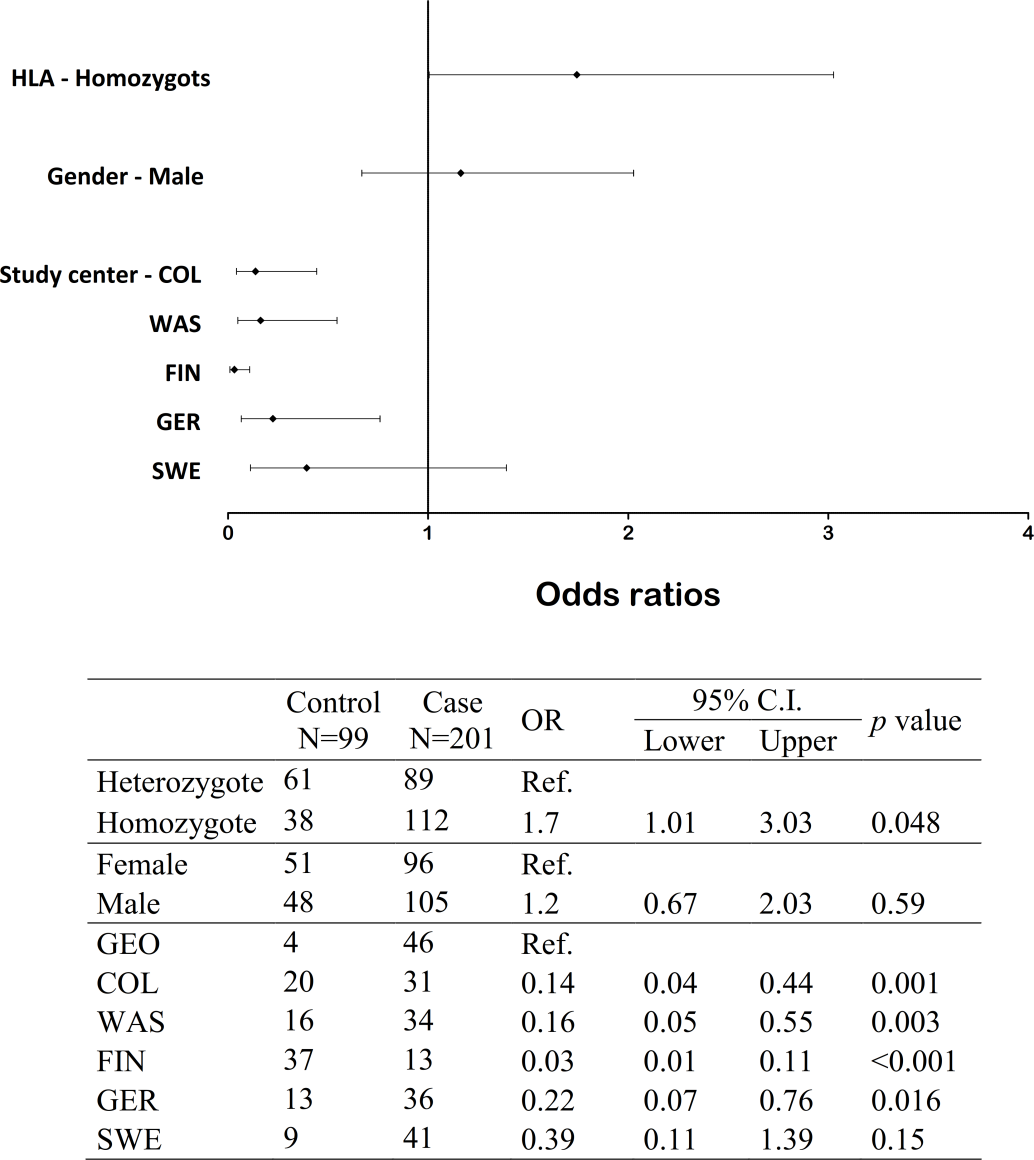
Forest plots showing the prediction of EV positivity by HLA-DQ, sex, and study center. A forest plot representation of the odds ratios with 95% confidence intervals in a model of predicting EV positivity by HLA-DQ homozygosity, sex, and study center is shown here. Heterozygosity, female sex, and Georgia were set as the reference groups, therefore they are missing from the graph.

65.3% of girls and 67.3% of boys were infected at least once with EVs (*p* = 0.54). The duration of the infection episodes did not differ between them either. However, the HLA genotype correlated with EV positivity, as children who were HLA-DQ2/8 heterozygous were less frequently EV positive compared to children who were homozygous for either HLA-DQ2 or HLA-DQ8 (*P* = 0.048; Fig 5 and S4 Fig).

## 5. Discussion

To our knowledge, this may be the largest study carried out so far to characterize EVs in otherwise healthy, young children in different countries, by analyzing stool samples which had been collected on a monthly basis and examined for the presence of EV RNA, followed by sequencing of an RT-PCR-amplified region of viral genome to identify the type of these EVs. The results clearly demonstrate the abundance of species A EVs in these children, a finding that is in contrast with the higher abundance of species B EVs reported in children with symptomatic and severe infections. For example, the Centers of Disease Control (CDC) reports that 85% of the top 15 prevalent strains of EVs belong to species B (see S5 Table) [21,22]. In addition, the present study points out the comparatively low frequency of all EV infections in Finland.

One important strength of our study is that the study subjects were not selected on the basis of symptoms or diseases. Thus, it provides information about the EVs circulating in young children of the general population, regardless of the symptoms and is only biased by the selection of certain HLA types. When combined with the data from hospitalized patients, this information will help to identify EVs with a high attack rate which are likely to cause clinically significant diseases (see S5 Table). For example, based on the CDC reports by The National Enterovirus Surveillance System (NESS) [23] CV-B group viruses (CV-B1 to 5) were detected in 34.2% (477/1394) of all reported EVs during the same years as our study was conducted (2006–2009) [1,21,22], while CV-Bs caused only 16.1% (N = 62) of EV infections diagnosed in the present study, being detected in 14.7% (N = 44) of the children. In contrast to the present study, reports by NESS are based on infections diagnosed through symptomatic disease voluntarily reported by participating laboratories since 1960s in the USA [23]. On the other hand, many of the EV-A types that were frequent in the present study (e.g. CV-A2, A4 and A5) are comparatively rare in hospitalized cases [1]. Comparison of our data to the reported NESS/CDC data was hampered by the infrequency of EV-A in the symptomatic patients. EV-A71 and CV-A16 are mostly reported as the causative agents of HFMD [24–27]. We identified 15 children with CV-A16 and 12 children with EV-A71 ranking 7^th^ and 10^th^ of all detected EVs, respectively. Interestingly, EV-D68 was not detected in any of our samples, despite its widespread circulation in the USA in 2014 [28]. However, one should be aware of factors, which may bias these comparisons. For example, CDC reports are based on varying virus detection methods and samples that have been collected from various anatomical sites and age groups. For example, the lack of respiratory samples may have led to an underestimation of the number of EVs that replicate predominantly in the respiratory track, including EV-D68 that was completely absent in the present study. In addition, since sample were collected regularly without extra samples taken when the child had symptoms of acute illness, the study probably underestimates the true frequency of EVs, and this makes it difficult to accurately assess the length of the infection episodes.

The observed abundance of the species A EVs is in line with previous studies carried out in similar child cohorts in Sweden and Finland [19,29]. Similarly, a German study (Babydiet) reported a surplus of EV-A compared to EV-B in healthy children with the highest prevalence of CV-A4, CV-A2, CV-A10 and E-25 [30] which also ranked 1st, 3rd, 5th, and 9th in our study. However, another study in Norway (MIDIA study) found EV-B types more frequently compared to EV-A types [31]. Altogether, these results suggest that species A EVs may be responsible for the majority of subclinical EV infections in children in Europe and in the USA. The five most frequently detected EV types were CV-A2, A4, A5, A6 and A10, representing altogether 53% of all identified EV infections. However, it is possible that the abundancy of different EV types may vary over time and may differ with the sample type analyzed (e.g. respiratory vs. fecal samples).

One additional strength of this study is that the samples were directly analyzed by highly sensitive RT-PCR without prior enrichment in cell cultures. This eliminated a possible under-representation of the EV types that do not replicate well in cell culture [7–9]. On the other hand, a potential weakness of the study was that the relatively low number of many of the individual serotypes reduced the statistical power for comparing their occurrence in different populations. In addition, more studies from other geographical regions are needed to obtain a better understanding of global EV circulation patterns. Further studies are also needed to evaluate the circulation of EVs in older age groups and during longer time periods.

Some viruses, including CV-A4, E-18, and E-25 had a longer shedding time than other EVs. This could be due to a less effective immunological response that fails to efficiently clear the infection or to other factors such as their strong tropism to the intestinal mucosa, higher replication efficiency, or other intrinsic viral factors. The shedding of EVs into stools can vary a lot and the longest reported shedding periods in immunocompetent children have ranged 6–11 weeks [32,33]. It was also reported that oral poliovirus type 3 vaccine strain can shed for as long as 100 days in individual vaccinees (34).

The low prevalence of EV infections in Finland is in line with our previous serological studies [34,35]. The reason for this phenomenon is not known but, hypothetically, it could be partly due to the cold climate—EVs circulate at low levels during the long winter season with subfreezing temperatures. This is supported by the seasonal pattern that is typical for EV infections [36]. A low prevalence of EV infections may also make young children more susceptible to a severe course of infection as previously described for poliovirus: The incidence of polio paralysis started to increase at the end of the 19^th^ century in western countries at the same time when the circulation of the three poliovirus types decreased. This change was related to a shift in the occurrence of the first infection to an older age, when maternal antibodies were no longer present in the children making them susceptible to the systemic spread of the virus and subsequent clinical symptoms [37,38]. Analogously, it has been speculated that the limited circulation of EVs could render Finnish children susceptible to EV infections and their spread to the pancreas, and thereby contribute to the high incidence of T1D in Finland [39].

Host factors modulate the course of many viral infections. For example, HLA genes have been shown to influence the progression of HIV infections and the severity of Hantaan virus infections [40–42]. T1D-associated HLA genes have also been reported to influence the immune response against EVs [43]. In the present study, the children who were heterozygous for HLA-DQ2 and -DQ8 had fewer EV infections than the children who were homozygous for these haplotypes. This is in line with other studies suggesting that homo- and heterozygosity in the HLA alleles may modulate the course of a viral infection [44,45]. It is possible that heterozygous individuals present a wider range of EV peptides to T-helper cells than homozygous individuals. This could result in a faster clearance of the infection and a stronger inflammatory response in heterozygous individuals compared to homozygous ones. On the other hand, we did not find any sex differences in EV positivity even though boys are known to be more prone to severe EV infections than girls. This may reflect the fact that the children of the current study were asymptomatic.

In conclusion, the present study shows that EVs are frequent causes of infections in young children with increased genetic risk for developing T1D. Viruses of the species *Enterovirus A* were the most frequent EVs detected in all geographic areas in this study. In addition, there is conspicuous variation between different geographic locations in the frequency of EV infections, and a particularly striking piece of evidence is showing the low frequency of EV infections in Finland. Such differences in the population dynamics of EVs may have important consequences for the severity of EV diseases (and T1D) in these populations.

## Supporting information

S1 Table Distribution of HLA-DQ genotypes across the study centers. The numbers show the number of children and the percentage within the center is also shown between brackets N# (%).
(PDF)

S2 Table Seasonal distribution of stool samples in different study centers according to the month of sample collection (% of all samples). (PDF)

S3 Table Oligonucleotides (primers and probes) employed in the EV RT-PCR. (PDF)

S4 Table Genotypes of EVs causing each individual infection episode in each study center and in US and European (EU) centers combined. (PDF)

S5 Table The most frequently reported EVs in CDC reports representing the years covered by the present study. The data has been extracted from the CDC reported Enterovirus and Human Parechovirus Surveillance in the USA, covering the years 2006–2009 when the samples were collected for the present study. The figures show the 16 most frequent EVs and parechoviruses in CDC reports, their rank and proportion (%) of all EV and parechovirus reports. The rank and proportion of the same viruses in the present study is shown for comparison. CDC reports were based on a total of 1395 typed EVs detected in different laboratories participating in The National Enterovirus Surveillance System (NESS) [21,22].
(PDF)

S6 Table Prevalence of different EV types by year of sample collection and age of the child. The number of EV types detected in each year and by the age of the child. The age has been divided into 6-month periods starting from birth.
(PDF)

S1 Fig Defining enterovirus infection episodes. EV infection episodes were defined by the presence of a positive PCR result, shown as the sample positivity in the figure, and the presence of the same virus in consecutive samples. In this example the infection episode includes two consecutive positive samples with the same virus identified by sequencing. The start of the episode (shown as S) was calculated as the mid-way between the time of the first positive sample (sample B in the figure) and the preceding sample (sample A in the figure), indicated as (B-A)/2. The end of the episode (shown as E) was defined as the mid-way point between the last of the consecutively positive samples with the same virus (sample C) and the first sample negative or positive for a different virus (sample D) indicated as (D-C)/2. The calculated mid-way point at the beginning and the end of the episode could not be more than 15 days. In case a Ct-value was not more than 25 for a positive sample at the beginning of an episode, the starting date was set at 10 days prior to the time of the first positive sample (shown as S´). In case a Ct-value was more than 35 for a positive sample at the end of the episode, the end date was set at 7 days after the time of the last positive sample (shown E´).
(PDF)

S2 Fig Distribution of Ct values of qPCR for EVs in stool samples. The Ct values are the average of 3 independent experiments.
(PDF)

S3 Fig Prevalence (%) of enteroviruses in children at different ages. (PDF)

S4 Fig EV positivity in children with different HLA-DQ genotypes and in boys and girls (per sample and per child). Logistic regression was carried out to further analyze the differences between HLA groups along with the effect of the sex and the study center (see Fig 5).
(PDF)
