# Supplementary material for: Molecular epidemiology of enteroviruses in young children at increased risk of type 1 diabetes
Source: PLoS One. 2018 Sep 7;13(9):e0201959. doi: 10.1371/journal.pone.0201959 (PMC6128458; doi:10.1371/journal.pone.0201959)
Supplement: S1 Table — The numbers show the number of children and the percentage within the center is also shown between brackets N# (%). (PDF) [file pone.0201959.s001.pdf]

**S1 Table. Distribution of HLA-DQ genotypes across the study centers.**

The numbers show the number of children and the percentage within the center is also shown between brackets N# (%).

| HLA | COL | GEO | WAS | FIN | GER | SWE | Total |
|---|---|---|---|---|---|---|---|
| DQ2/2 | 10 (20) | 15 (30) | 23 (46) | 4 (8) | 19 (39) | 10 (20) | 81 (27) |
| DQ2/8 | 28 (55) | 23 (46) | 18 (36) | 34 (68) | 21 (43) | 26 (52) | 150 (50) |
| DQ8/8 | 13 (26) | 12 (24) | 9 (18) | 12 (24) | 9 (18) | 14 (28) | 69 (23) |
| Total | 51 (100) | 50 (100) | 50 (100) | 49 (100) | 50 (100) | 50 (100) | 300 (100) |
