# Supplementary material for: Molecular epidemiology of enteroviruses in young children at increased risk of type 1 diabetes
Source: PLoS One. 2018 Sep 7;13(9):e0201959. doi: 10.1371/journal.pone.0201959 (PMC6128458; doi:10.1371/journal.pone.0201959)
Supplement: S2 Table — (PDF) [file pone.0201959.s002.pdf]

S2 Table. Seasonal distribution of stool samples in different study centers according to the month of sample collection (% of all samples).

	Center						
Month	COL	GEO	WAS	FIN	GER	SWE	Total
Jan.	9.5	9.6	9.6	8.7	9.6	8.9	9.3
Feb.	8.5	8.8	8.6	8.0	8.1	7.9	8.3
Mar.	8.8	9.0	8.0	8.4	9.6	8.8	8.7
Apr.	8.2	7.2	9.5	7.5	9.1	8.9	8.4
May.	8.5	9.1	9.0	9.3	9.1	8.9	9.0
Jun.	8.3	7.8	8.6	8.7	8.5	6.8	8.1
Jul.	8.6	7.6	9.0	8.7	7.0	8.3	8.2
Aug.	7.9	8.8	7.4	8.4	6.4	7.3	7.7
Sep.	8.5	6.9	4.8	7.7	7.0	7.4	7.0
Oct.	8.0	8.5	9.5	8.3	9.0	9.5	8.8
Nov.	7.9	9.7	8.6	8.7	8.8	8.8	8.7
Dec.	7.6	6.9	7.7	7.7	7.8	8.3	7.6
Total	100	100	100	100	100	100	100
