# Supplementary material for: Molecular epidemiology of enteroviruses in young children at increased risk of type 1 diabetes
Source: PLoS One. 2018 Sep 7;13(9):e0201959. doi: 10.1371/journal.pone.0201959 (PMC6128458; doi:10.1371/journal.pone.0201959)
Supplement: S3 Table — (PDF) [file pone.0201959.s003.pdf]

S3 Table. Oligonucleotides (primers and probes) employed in the EV RT-PCR.

Oligonucleotide	Sequence
Forward primer	CGG CCC CTG AAT GCG GCT AA
Reverse primer	GAA ACA CGG ACA CCC AAA GTA
Probe #1	FAM-TCT GTG GCG GAA CCG ACT A-TAMRA
Probe #2	FAM-TCT GCA GCG GAA CCG ACT A-TAMRA
