# Supplementary material for: Molecular epidemiology of enteroviruses in young children at increased risk of type 1 diabetes
Source: PLoS One. 2018 Sep 7;13(9):e0201959. doi: 10.1371/journal.pone.0201959 (PMC6128458; doi:10.1371/journal.pone.0201959)
Supplement: S4 Table — (PDF) [file pone.0201959.s004.pdf]

S4 Table. Types of EVs causing each individual infection episode in each study center and in US and European (EU) centers combined.

		Number of infection episodes									
Species	Serotypes	USA	COL	GEO	WAS	EU	FIN	GER	SWE	Total	% of typed
EV-A	CV-A2	27	8	15	4	7	1	2	4	34	10.8
	CV-A4	31	14	8	9	17	0	9	8	48	15.3
	CV-A5	14	1	5	8	11	0	6	5	25	8.0
	CV-A6	24	5	9	10	15	1	8	6	39	12.4
	CV-A8	2	0	2	0	2	0	1	1	4	1.3
	CV-A10	12	1	7	4	9	0	5	4	21	6.7
	CV-A16	10	2	6	2	5	0	4	1	15	4.8
	EV-A71	3	0	2	1	8	0	6	2	11	3.5
	Subtotal	123	31	54	38	74	2	41	31	197	62.7
EV-B	CV-A9	4	0	4	0	5	0	4	1	9	2.9
	CV-B1	11	5	3	3	2	0	0	2	13	4.1
	CV-B2	7	1	5	1	1	0	1	0	8	2.5
	CV-B3	5	2	2	1	6	0	3	3	11	3.5
	CV-B4	6	3	2	1	8	3	0	5	14	4.5
	CV-B5	4	0	4	0	3	0	3	0	7	2.2
	E-3	2	0	2	0	0	0	0	0	2	0.6
	E-6	3	0	2	1	3	0	2	1	6	1.9
	E-9	1	0	0	1	3	0	1	2	4	1.3
	E-11	4	2	1	1	7	0	3	4	11	3.5
	E-13	2	0	2	0	4	0	1	3	6	1.9
	E-18	7	3	2	2	1	0	0	1	8	2.5
	E-21	0	0	0	0	1	0	1	0	1	0.3
	E-25	8	1	6	1	1	0	1	0	9	2.9
	E-30	3	0	2	1	3	0	1	2	6	1.9
	Sub total	67	17	37	13	48	3	21	24	115	36.6
EV-C	CV-A1	0	0	0	0	1	0	1	0	1	0.3
	CV-A22	1	0	1	0	0	0	0	0	1	0.3
	Sub total	1	0	1	0	1	0	1	0	2	0.6
Episodes with known serotypes		191	48	92	51	123	5	63	55	314	100.0
Episodes with unknown serotypes		58	17	22	19	36	9	14	13	94	NA
Total episodes		249	65	114	70	159	14	77	68	408	NA
