# Supplementary material for: Molecular epidemiology of enteroviruses in young children at increased risk of type 1 diabetes
Source: PLoS One. 2018 Sep 7;13(9):e0201959. doi: 10.1371/journal.pone.0201959 (PMC6128458; doi:10.1371/journal.pone.0201959)
Supplement: S5 Table — The data has been extracted from the CDC reported Enterovirus and Human Parechovirus Surveillance in the USA, covering the years 2006–2009 when the samples were collected for the present study. The figures show the 16 most frequent EVs and parechoviruses in CDC reports, their rank and proportion (%) of all EV and parechovirus reports. The rank and proportion of the same viruses in the present study is shown for comparison. CDC reports were based on a total of 1395 typed EVs detected in different laboratories participating in The National Enterovirus Surveillance System (NESS) [21,22]. (PDF) [file pone.0201959.s005.pdf]

S5 Table. The most frequently reported EVs in CDC reports representing the years covered by the present study.

The data has been extracted from the CDC reported enterovirus and human parechovirus surveillance in the USA, covering the years 2006-2009 when the samples were collected for the present study. The figures show the 16 most frequent EVs and parechoviruses in CDC reports, their rank and proportion (%) of all EV and parechovirus reports. The rank and proportion of the same viruses in the present study is shown for comparison. CDC reports were based on a total of 1395 typed EVs detected in different laboratories participating in The National Enterovirus Surveillance System (NESS) [1, 2].

CDC report (N=1395)				EV species	Present study (N=384)		
Serotype	Rank	%	N		Rank	%	N
CV-B1	1	15.2	212	B	8	3.7	14
E-9	2	10.1	140	B	15	1.0	4
E-6	3	9.8	136	B	12	2.6	10
E-18	4	8.3	115	B	10	3.1	12
E-30	5	7.1	99	B	14	2.1	8
CV-B4	6	7.0	97	B	6	4.2	16
CV-A9	7	7.0	97	B	11	2.9	11
E-11	8	5.2	72	B	9	3.4	13
CV-B3	9	4.8	66	B	9	3.4	13
CV-B5	10	4.2	58	B	13	2.3	9
EV-D68	11	3.4	47	D	-	0	0
CV-B2	12	3.1	43	B	12	2.6	10
E-25	13	2.0	27	B	9	3.4	13
E-7	14	1.7	23	B	-	0	0
Human parechovirus 1	15	1.5	21	-	N.T.	N.T.	N.T.
CV-A16	16	1.4	20	A	7	3.9	15
Total	-	91.3	1273	-	-	38.5	

N.T. = not tested
