# Supplementary material for: Molecular epidemiology of enteroviruses in young children at increased risk of type 1 diabetes
Source: PLoS One. 2018 Sep 7;13(9):e0201959. doi: 10.1371/journal.pone.0201959 (PMC6128458; doi:10.1371/journal.pone.0201959)
Supplement: S6 Table — The number of EV types detected in each year and by the age of the child. The age has been divided into 6-month periods starting from birth. (PDF) [file pone.0201959.s006.pdf]

S6 Table. Prevalence of different EV types by year of sample collection and age of the child.

The number of EV types detected in each year and by the age of the child. The age has been divided into 6-month periods starting from birth.

	Study years					Age divided in 6 month periods				
	2005	2006	2007	2008	2009	6	12	18	24	Total
CVA1	0	1	0	0	0	0	0	0	1	1
CVA2	1	7	17	7	0	3	12	8	9	32
CVA4	2	4	29	16	1	5	19	12	16	52
CVA5	3	4	6	13	0	3	9	8	6	26
CVA6	1	13	6	20	0	2	16	10	12	40
CVA8	0	2	1	1	0	0	2	1	1	4
CVA9	3	4	1	1	0	2	5	2	0	9
CVA10	0	6	3	12	0	1	3	7	10	21
CVA16	0	4	7	3	0	3	4	4	3	14
CVA22	0	0	0	0	1	0	0	0	1	1
CVB1	0	0	8	5	0	1	7	1	4	13
CVB2	1	5	2	0	0	0	3	3	2	8
CVB3	1	4	2	4	0	2	2	3	4	11
CVB4	1	0	7	2	0	0	6	2	2	10
CVB5	1	0	7	0	0	0	3	2	3	8
E3	0	0	0	2	0	1	1	0	0	2
E6	0	2	3	1	0	1	3	1	1	6
E9	1	0	3	0	0	2	1	0	1	4
E11	0	5	4	2	0	0	4	2	5	11
E13	0	4	2	0	0	0	3	1	2	6
E18	0	1	4	3	1	1	1	2	5	9
E21	1	0	0	0	0	0	1	0	0	1
E25	3	0	0	5	1	0	3	0	6	9
E30	0	2	2	2	0	1	0	2	3	6
EV-A71	0	4	6	1	0	0	4	5	2	11
Total	19	72	120	100	4	28	112	76	99	315
