# Supplementary material for: Molecular epidemiology of enteroviruses in young children at increased risk of type 1 diabetes
Source: PLoS One. 2018 Sep 7;13(9):e0201959. doi: 10.1371/journal.pone.0201959 (PMC6128458; doi:10.1371/journal.pone.0201959)

S1 Fig. Defining enterovirus infection episodes.

EV infection episodes were defined by the presence of a positive PCR result, shown as the sample positivity in the figure, and the presence of the same virus in consecutive samples. In this example the infection episode includes two consecutive positive samples with the same virus identified by sequencing. The start of the episode (shown as S) was calculated as the mid-way between the time of the first positive sample (sample B in the figure) and the preceding sample (sample A in the figure), indicated as (B-A)/2. The end of the episode (shown as E) was defined as the mid-way point between the last of the consecutively positive samples with the same virus (sample C) and the first sample negative or positive for a different virus (sample D) indicated as (D-C)/2. The calculated mid-way point at the beginning and the end of the episode could not be more than 15 days. In case a Ct-value was not more than 25 for a positive sample at the beginning of an episode, the starting date was set at 10 days prior to the time of the first positive sample (shown as S´). In case a Ct-value was more than 35 for a positive sample at the end of the episode, the end date was set at 7 days after the time of the last positive sample (shown E´).

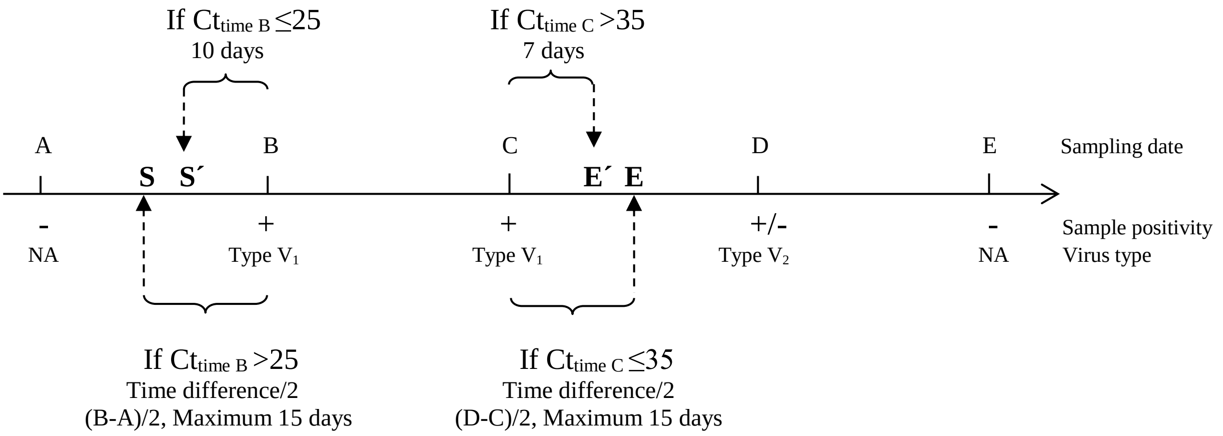

Supplement: S1 Fig — EV infection episodes were defined by the presence of a positive PCR result, shown as the sample positivity in the figure, and the presence of the same virus in consecutive samples. In this example the infection episode includes two consecutive positive samples with the same virus identified by sequencing. The start of the episode (shown as S) was calculated as the mid-way between the time of the first positive sample (sample B in the figure) and the preceding sample (sample A in the figure), indicated as (B-A)/2. The end of the episode (shown as E) was defined as the mid-way point between the last of the consecutively positive samples with the same virus (sample C) and the first sample negative or positive for a different virus (sample D) indicated as (D-C)/2. The calculated mid-way point at the beginning and the end of the episode could not be more than 15 days. In case a Ct-value was not more than 25 for a positive sample at the beginning of an episode, the starting date was set at 10 days prior to the time of the first positive sample (shown as S´). In case a Ct-value was more than 35 for a positive sample at the end of the episode, the end date was set at 7 days after the time of the last positive sample (shown E´). (PDF) [file pone.0201959.s007.pdf]
