# Supplementary material for: Molecular epidemiology of enteroviruses in young children at increased risk of type 1 diabetes
Source: PLoS One. 2018 Sep 7;13(9):e0201959. doi: 10.1371/journal.pone.0201959 (PMC6128458; doi:10.1371/journal.pone.0201959)

S2 Fig. Distribution of Ct values of qPCR for EVs in stool samples.

The Ct values are the average of 3 independent experiments.

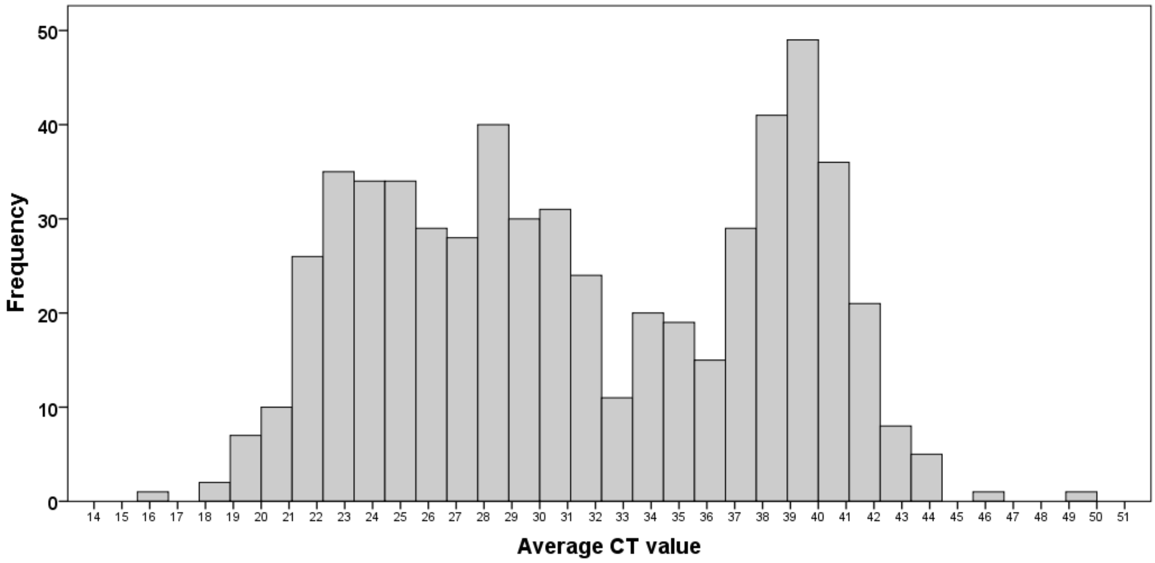

Supplement: S2 Fig — The Ct values are the average of 3 independent experiments. (PDF) [file pone.0201959.s008.pdf]
