# Supplementary figures and images for: Molecular epidemiology of enteroviruses in young children at increased risk of type 1 diabetes
Source: PLoS One. 2018 Sep 7;13(9):e0201959. doi: 10.1371/journal.pone.0201959 (PMC6128458; doi:10.1371/journal.pone.0201959)

**S3 Fig. Prevalence (%) of enteroviruses in children at different ages.**

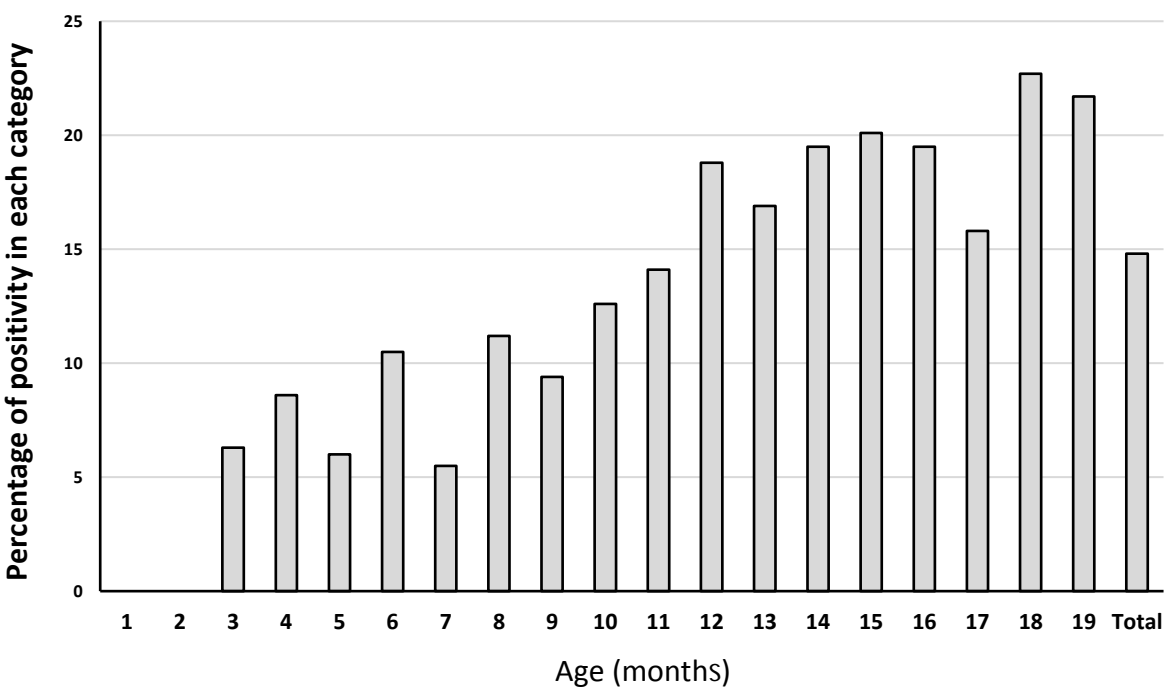

Supplement: S3 Fig — (PDF) [file pone.0201959.s009.pdf]
