# Supplementary material for: Molecular epidemiology of enteroviruses in young children at increased risk of type 1 diabetes
Source: PLoS One. 2018 Sep 7;13(9):e0201959. doi: 10.1371/journal.pone.0201959 (PMC6128458; doi:10.1371/journal.pone.0201959)

S4 Fig. EV positivity in children with different HLA-DQ genotypes and in boys and girls (per sample and per child).

Logistic regression was carried out to analyze the differences between HLA groups along with the effect of the sex and the study center (see Fig 5).

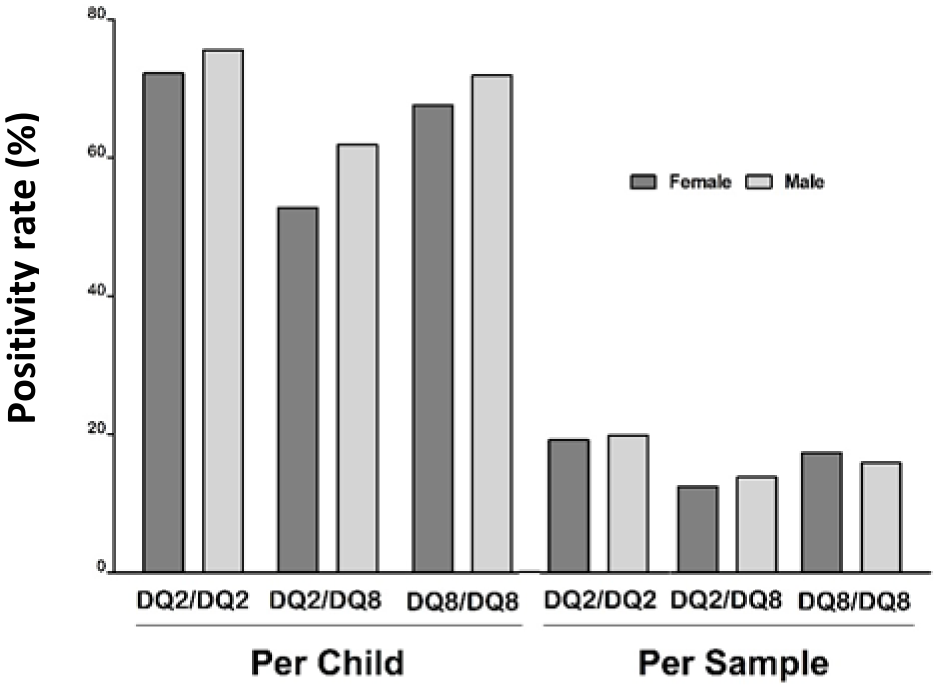

Supplement: S4 Fig — Logistic regression was carried out to further analyze the differences between HLA groups along with the effect of the sex and the study center (see Fig 5). (PDF) [file pone.0201959.s010.pdf]
